# The diversity of prokaryotes and fungi hosted in crude oils

**DOI:** 10.1128/spectrum.01689-24

**Published:** 2025-05-28

**Authors:** Xiaoxue Qi, Shijie Bai, Suyang Cai, Xuegong Li, Qilin Xiao

**Affiliations:** 1 College of Resources and Environment, Yangtze University 728177, Wuhan, Hubei, China; 2 Institute of Deep-sea Science and Engineering, Chinese Academy of Sciences 383875 , Sanya, Hainan, China; University of Minnesota Twin Cities, St. Paul, Minnesota, USA

**Keywords:** crude oil, microorganisms, microbial diversity, internal transcribed spacer, oil biodegradation, reservoir exploitation

## Abstract

The diversity of prokaryotes and fungi in crude oils has not been understood clearly, though unique microbial communities may be hosted in crude oil. This study investigated the chemical compositions and microbial communities of crude oils from Henan, Bamianhe, and Jianghan oilfields of China. Statistical analysis revealed significant variations of both prokaryotic and fungal communities (*P* < 0.05) within different oilfields and oils with different biodegradation levels. Diversity analysis showed little difference in prokaryotic, but a significant difference in fungal (*P* < 0.05). Prokaryotic diversity was higher in heavily biodegraded oils than those in unaltered and slightly biodegraded oils; the opposite was true for fungal diversity (*P* < 0.05). Moreover, thermophilic prokaryotes were detected mainly in biodegraded heavy oils produced by the practice of thermal recovery from Henan and Bamianhe oilfields, and halophilic prokaryotes were detected mainly in oils from sandstone reservoirs containing hypersaline formation water from Jianghan Oilfield. Accordingly, microbial communities in oils are affected by oil biodegradation, extraction practices, and natural environments of native inhabitants in subsurface petroleum reservoirs.

**IMPORTANCE:**

The biological activities of endogenous microorganisms in crude oil play an important role in the production and development of crude oil. Although there have been many microbiological investigations of crude oil-contaminated sites, our understanding of the phylogenetic diversity, metabolic capabilities, and community dynamics of microbial communities within crude oil is far from complete. In this paper, the prokaryotic and fungal communities of three oil fields in different regions of China were analyzed, and several factors affecting microbial degradation were further identified. This study provides a new direction for the subsequent investigation of microbial activities inside crude oil.

## INTRODUCTION

As one of the unique deep subsurface ecosystems, petroleum reservoirs can host various microorganisms; these microorganisms can survive by consuming organic molecules in crude oil and hence have a significant impact on the chemical composition and physical properties of crude oil and thus the economic values of petroleum reservoirs (1). Oils altered by microorganisms are usually depleted in aliphatic compounds and enriched in NSO-heterocyclic compounds compared to the unaltered oils (2), resulting in the formation of biodegraded heavy oils. Specific microbial organisms can metabolize hydrocarbon molecules to produce intermediates that act as chemical surfactants, reducing oil viscosity and enhancing oil recovery (3, 4). On the contrary, sulfate-reducing bacteria in petroleum reservoirs can produce toxic and corrosive hydrogen sulfide, which can react with water-soluble metals to form insoluble metal sulfides. This significantly increases the risks to health, safety, and environment during petroleum exploration and development, and may also reduce reservoir porosity and permeability (5, 6).

However, most published studies have focused mainly on either oil-contaminated soil and water on the Earth surface (7, 8) or on the formation water produced from subsurface reservoirs (9, 10). Few published studies concentrate on microbial communities within crude oil itself, though unique prokaryotes and fungi can be hosted in crude oil (11–14). Unique abundant information about microbial communities, therefore, has been erased unintentionally. Yoshida et al. 11 cultured and then detected typical microorganisms in crude oil including *Ochrobactrum anthropi*, *Burkholderia cepacia*, *Stenotrophomonas maltophilia*, *Propionibacterium acnes*, and *Brevundimonas diminuta*. However, the culturable microorganisms may not represent well the original microbial community in crude oil (11). In order to confirm this assumption, Yamane et al. 12 investigated the microbial communities in crude oils from the Middle East, Japan, and China. The experimental results demonstrated that *Acinetobacter*, *Propionibacterium*, *Sphingobium*, and *Bacillales* are the most prevalent microorganisms in oils from the Middle East. In contrast, abundant *Thermophilic* and *Clostridia* bacteria were detected in oils from China and Japan (12). Liu et al. 13 collected crude oils and associated water samples from an oil production plant. The experimental results demonstrated that *Burkholderia* sp., *Brevundimonas* sp., *Propionibacterium* sp., *Ochrobactrum* sp., and *Stenotrophomonas* sp. are the common bacterial groups in crude oil. *Hippea* sp., *Acidovorax* sp., *Arcobacter* sp., *Pseudomonas* sp., *Thiomicrospira* sp., *Brevibacterium* sp., *Tissierella* sp., and *Peptostreptococcus* sp. are more abundant in water samples (13). Moreover, the indigenous microbial communities in petroleum reservoirs can be artificially altered through oil recovery technologies, such as water-flooding and cyclic steam injection, which modify the natural environment of petroleum reservoirs (15).

Previous investigations have also demonstrated the important role of fungi in degrading petroleum hydrocarbons, though these species were not originally isolated from crude oil (16, 17). EL-Hanafy et al. showed that the *Aspergillus* and *Penicillium* isolated from oil-contaminated soil samples in the Yanbu region near the Red Sea are highly effective in degrading crude oil (18). Al-Hawash et al. (19) demonstrated that two fungal strains (*Penicillium* strains RMA1 and RMA2) isolated from a petroleum-polluted soil sample in the Rumaila Oilfield are capable of using crude oil as carbon and energy sources for growth. Alharbi et al. revealed that *Aspergillus terreus* KC462061 isolated from the roots of date palm (20) plays a critical role in oil biodegradation (21). However, the abovementioned fungi communities are extracted mainly from the surface soil samples, and our knowledge regarding the indigenous fungi in petroleum reservoirs is relatively limited.

In order to evaluate the diversity of prokaryotes and fungi hosted in crude oils, 14 oil samples were collected from the Henan, Jianghan, and Bamianhe oilfields of China. Henan Oilfield in Henan Province produces typical biodegraded heavy oil by using thermal recovery technology (22). Jianghan Oilfield in Hubei Province is located in a typical hypersaline lacustrine basin and produces sour crude oils (23). Bamianhe Oilfield in Shandong Province is situated in the Dongying Depression of the Bohai Bay basin and produces normal oil in the northern area and biodegraded heavy oil in the southern area by using thermal recovery technology similar to Henan Oilfield (24). This provides us a good opportunity to decipher the microbial communities in crude oils with different biodegradation levels, native environments of petroleum reservoirs, and artificial alteration through steam-assisted gravity drainage conducted on heavy oil sands.

By using gas chromatography-mass spectrometry (GC-MS) and Illumina high-throughput sequencing measurements together, we (i) reveal the molecular compositions of collected oils and biodegradation levels; (ii) decipher the shared and unique prokaryotes and fungi within these oils; and (iii) evaluate the environmental parameters regulating the endogenous microbial community of crude oil. This should improve our understanding of the diversity of endogenous prokaryotic and fungal communities in petroleum reservoirs and hence oil daily productivity by microbial enhanced oil recovery.

## MATERIALS AND METHODS

### Sampling

**Fig 1:**
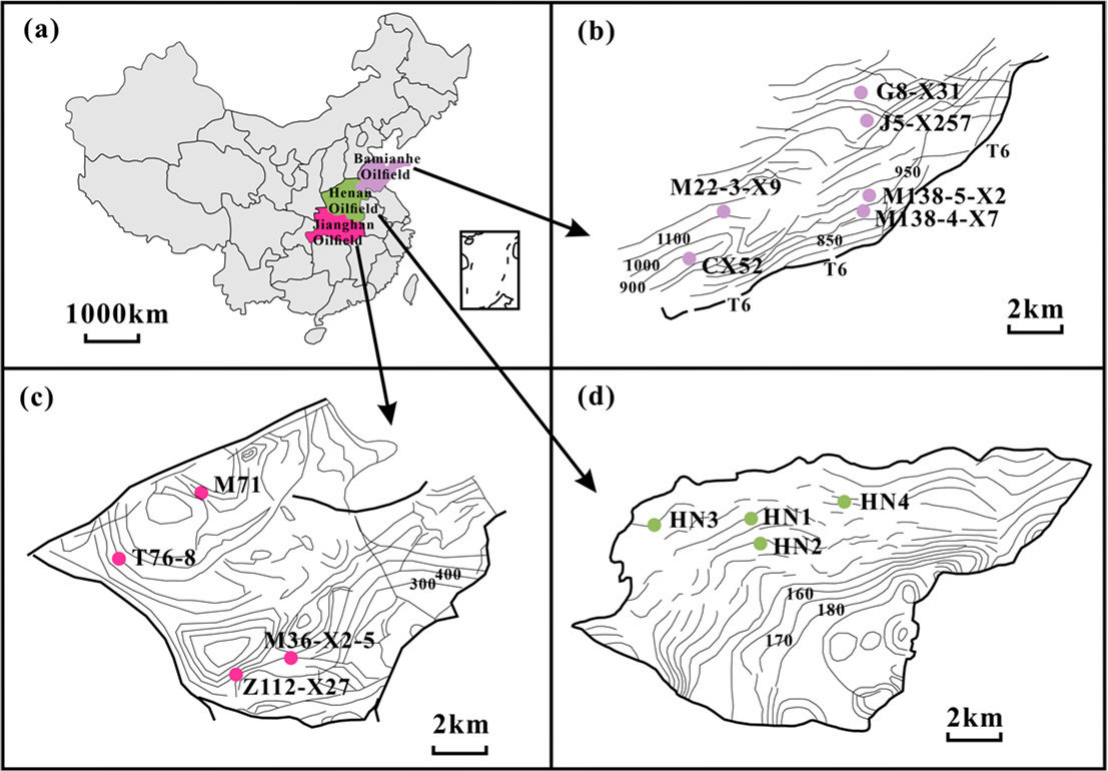
Distribution maps of oil samples from three oilfields. (a) Three oilfields; (b) Bamianhe Oilfield; (c) Jianghan Oilfield; and (d) Henan Oilfield.

Fourteen oil samples were collected from the well heads in three oilfields in China (Fig. 1; Table 1). Specifically, four samples, HN1–HN4, were collected from the Eocene Hetaoyuan Formation (E_h3_) in Henan Oilfield. Six oil samples were collected from the Eocene Shahejie Formation (Es_4_) in Bamianhe Oilfield. Four oil samples were collected from the Eocene Qianjiang (Eq) and Xingouzui Formation (Ex) in Jianghan Oilfield. All samples were stored at 4°C in the refrigerator for further geochemical and biological measurements.

**TABLE 1:** The geochemical characteristics of the samples, including the formations and depths of wells, and the molecular compositions and associated parameters of collected oil samples^*a*^

Oilfield	Henan				Bamainhe						Jianghan			
Well	HN1	HN2	HN3	HN4	CX52	M138-5-X2	M22-3-X9	M138-4-X7	J5-X257	G8-X31	M71	Z112-X27	T76-8	M36-X2-5
Formation	E_h3_	E_h3_	E_h3_	E_h3_	Es_4_	Es_4_	Es_3_	Es_4_	Es_3_	Es_4_	Ex	Eq_1_	Eq_4_	Ex
Burial depth (m)	578	580	584	590	1,029	1,081	1,089	1,163	1,355	2,403	877	1,098.5	1,226	1,407.5
Reservoir temperature (°C)	38.1	38.2	38.4	38.6	52.0	54.6	55.0	58.7	68.3	120.7	60.1	55.1	58.5	63.3
CPI	/	/	/	/	/	/	/	/	/	0.96	1.01	0.80	0.84	1.01
Σ*n-*C_21−_/Σ*n-*C_22+_	/	/	/	/	/	/	/	/	/	0.49	0.43	0.75	0.48	0.87
Pr/Ph	0.00	0.00	0.00	0.00	0.16	0.17	0.00	0.16	0.00	0.28	0.48	0.27	0.19	0.46
Ga/C_30_H	0.47	0.50	0.47	0.46	1.03	1.05	0.86	1.04	0.76	0.57	0.88	1.23	1.33	0.58
C_19_–C_23_TT/C_30_H	0.11	0.09	0.13	0.10	0.06	0.07	0.08	0.07	0.06	0.10	0.77	0.14	0.10	0.56
Ts/(Ts+Tm)	0.17	0.16	0.18	0.17	0.24	0.26	0.32	0.25	0.29	0.41	0.47	0.23	0.15	0.39
C_29_ ααα 20 S/(20S + 20R)	0.38	0.35	0.30	0.34	0.29	0.28	0.31	0.28	0.28	0.38	0.50	0.39	0.38	0.48
C_29_ ββ/(αα+ββ)	0.31	0.32	0.30	0.31	0.28	0.28	0.31	0.28	0.29	0.34	0.56	0.39	0.33	0.51
20R-ααα C_27_ (%)	12.21	11.27	13.11	12.50	19.78	21.18	18.72	21.35	20.10	14.01	5.67	18.99	21.24	6.74
20R-ααα C_28_ (%)	13.00	14.21	13.21	14.13	12.87	13.44	12.33	13.38	12.56	9.98	6.36	10.35	11.61	7.10
20R-ααα C_29_ (%)	19.97	19.23	18.67	18.29	19.51	20.80	20.03	20.62	21.27	17.96	10.63	14.57	17.18	12.95
PM	4–5	4–5	4–5	4–5	4–5	4–5	4–5	4–5	4–5	0	1–2	0	1–2	0

^*a*^ Pr: pristane, Ph: phytane, Ts: 18α(H)-22,29,30-trinorneohopane (C27H46), Tm: 17α(H)-22,29,30-trinorhopane (C27H46) PM: The schemes for classifying the degree of biodegradation by Peters and Moldowan (25). The extent of biodegradation of mature crude oil can be ranked on a scale of 1–10 based on differing resistance of compound classes to microbial attack. CPI={(C25+C27+C29+C31+C33) [1/(C24+C26+C28+C30+C32) + 1/(C26+C28+C30+C32+C34)]}/2 604 “/”: No Date available.

### GC

The gas chromatographic analysis of whole oil was carried out by using an Agilent 7890B gas chromatograph equipped with a 50 m × 0.32 mm i.d. fused silica capillary column covered with a 0.40 m film of CP-SIL5CB and a flame ionization detector. The split injector system was used with a split ratio of 50:1. Nitrogen was the carrier gas with 1.0 mL/min. The temperature program was 35°C for 15 min, heating at 4°C/min to 310°C, and holding at 310°C for 30 min (26).

### GC-MS

Asphaltenes were removed by precipitation with *n*-hexane followed by filtration column chromatography with aluminum oxide as the stationary phase was used to separate the deasphaltened oil into saturated, aromatic, and resins fractions with *n*-hexane, benzene, and a combination of dichloromethane and methanol (9:1, vol/vol) as the eluents. The saturated fraction was used for the GC-MS measurements.

The GC-MS was equipped with a Hewlett–Packard 6890 gas chromatograph interfaced to a Micromass Platform II spectrometer. The HP 5 MS column (30 m × 0.25 mm i.d × 0.25 µm) was utilized to do the separation. Followed by 2 min at 60°C, the temperature was raised to 315°C at a rate of 3°C per minute, with a 15-min holding period. Helium served as the carrier gas, and a 1.0 mL/min flow rate was established. The ion source was at 200°C and the transfer line was at 250°C. The electron ionization mode at 70 eV was the ion source’s operating state. The full scan and SIM GC-MS techniques were used to identify the targeted compounds. In the full scan GC-MS study, the mass range was 50–550 Da, and the scan time was 1 s (26). The selected ions of SIM GC-MS analysis were *m/z* 85 for *n*-alkanes, *m/z* 191 for terpanoids, and *m/z* 217 for steranes.

### DNA extraction

A total of 15 g of crude oil was mixed with the equal volume of isooctane (2,2,4-trimethylpentane) at 4°C. The mixed sample was centrifuged at 4°C for 5,000 × *g* for 60 min, then the precipitate was suspended in 15 mL isooctane for 30 min at 4°C for 5,000 × *g*, and then suspended in 10 mL isooctane for three consecutive times, and centrifuged at 4°C for 5,000 × *g* for 30 min. Finally, the precipitate was vacuum freeze-dried (11, 12). Then, DNA was extracted from each dried sample (ca. 250 mg) using the DNeasy PowerSoil Pro Kit (QIAGEN, Germantown, MD, USA).

### PCR amplification and sequencing

The extracted DNA was utilized for amplification of the V4 region of the 16S rRNA gene using the primer pairs 515f Modified (5′-GTGYCAGCMGCCGCGGTAA-3′) and 806r Modified (5′-GGACTACNVGGGTWTCTAAT-3′) (27). The 515f-806r primer pair was shown to be biased against environmental archaea (28), but the primer pairs 515f Modified (5′-GTGYCAGCMGCCGCGGTAA-3′) and 806r Modified (5′-GGACTACNVGGGTWTCTAAT-3′) improve detection of archaea, while maintaining original amplification efficacy for previously detectable taxa, as validated through mock community analyses (27). Numerous literatures have reported the efficacy of the primer pairs 515f Modified (5′-GTGYCAGCMGCCGCGGTAA-3′) and 806r Modified (5′-GGACTACNVGGGTWTCTAAT-3′) in recovering archaeal diversity (29, 30). The extracted DNA was quantified by a Qubit fluorometer (Invitrogen Inc. Manufacturer: Life Technologies Holdings Pte Ltd., Singapore). The 16S rRNA gene PCR cycling conditions were as follows: denaturation at 95°C for 3 min, followed by 27 cycles at 95°C for 30 s, 55°C for 30 s, 72°C for 45 s, and a final extension at 72°C for 10 min. Furthermore, these samples were subjected to ITS sequencing to investigate the fungal communities present in the crude oil samples. Primer pairs ITS1F (5′-CTTGGTCATTTAGAGGAAGTAA-3′) and ITS2R (5′-GCTGCGTTCTTCATCGATGC-3′) (31) were selected for ITS sequencing. The samples DNA amplification conditions were as follows: denaturation at 95°C for 3 min, followed by 35 cycles at 95°C for 30 s, 55°C for 30 s, 72°C for 45 s, and a final extension at 72°C for 10 min. Triplicate PCR products were combined after purification using a TaKaRa purification kit (TaKaRa, Kusatsu, Shiga, Japan). The sample DNA was processed by the TruSeq DNA sample preparation kit (Illumina, San Diego, CA, USA) in accordance with the manufacturer’s instructions for library construction. The libraries were sequenced with a paired-end 300 bp sequence read run on the HiSeq platform (Illumina) at MajorBio Co. Ltd. in Shanghai, China. Raw sequencing reads of all samples were deposited in the NCBI database (http://www.ncbi.nlm.nih.gov/) under the BioProject accession numbers PRJNA1020134 for the prokaryotic community data sets and PRJNA1020147 for the fungal community data sets.

### Microbial community analysis

Barcodes and forward and reverse primers (one mismatch each allowed) were removed after allocating sequences to the appropriate samples to obtain clean data. The average fragment length for prokaryotes was 253 bp and for fungi, it was 255 bp. Pair-end sequences of appropriate length with at least a 30 bp overlap were obtained using the FLASH program (v.1.2.8) (32). Used the Btrim program (v.0.2.0) to retain high-quality sequences without ambiguous bases (NS) and used sequences with prokaryotes length from 245 to 260 bp and fungi length from 230 to 280 bp for additional analyses (33). The amplicon sequence variants (ASVs) of the default settings were generated by UNOISE3 (34). The sequences of bacterial, archaeal, and fungal were compared with the SILVA 138 database (35). A representative sequence from each ASV of the 16S rRNA gene sequencing was chosen for taxonomic annotation, and the representative sequence of each ASV in the ITS gene sequence was annotated by comparison with the Unite database (v.8.3) (36). The unique and common ASVs of prokaryotes and fungi in each group were analyzed according to the conditions of two kinds of degradation degree and three regions. Considered the different sequencing depths, in order to standardize the reads of each sample, the ASVs were randomly resampled. The statistical study of the α-diversity indices (Shannon, Inverse Simpson, and Chao1) and observed richness was used to estimate the diversity of prokaryotic and fungal communities with different degradation degrees and different regions (37). The Mothur software (38) and R language version 4.1.3 (39) were used to generate these α-diversity indices. The differences in prokaryotic and fungal community structure were examined using non-metric multidimensional scaling (NMDS) and statistical methods based on ß-diversity.

## RESULTS

### Molecular compositions of crude oils

**Fig 2:**
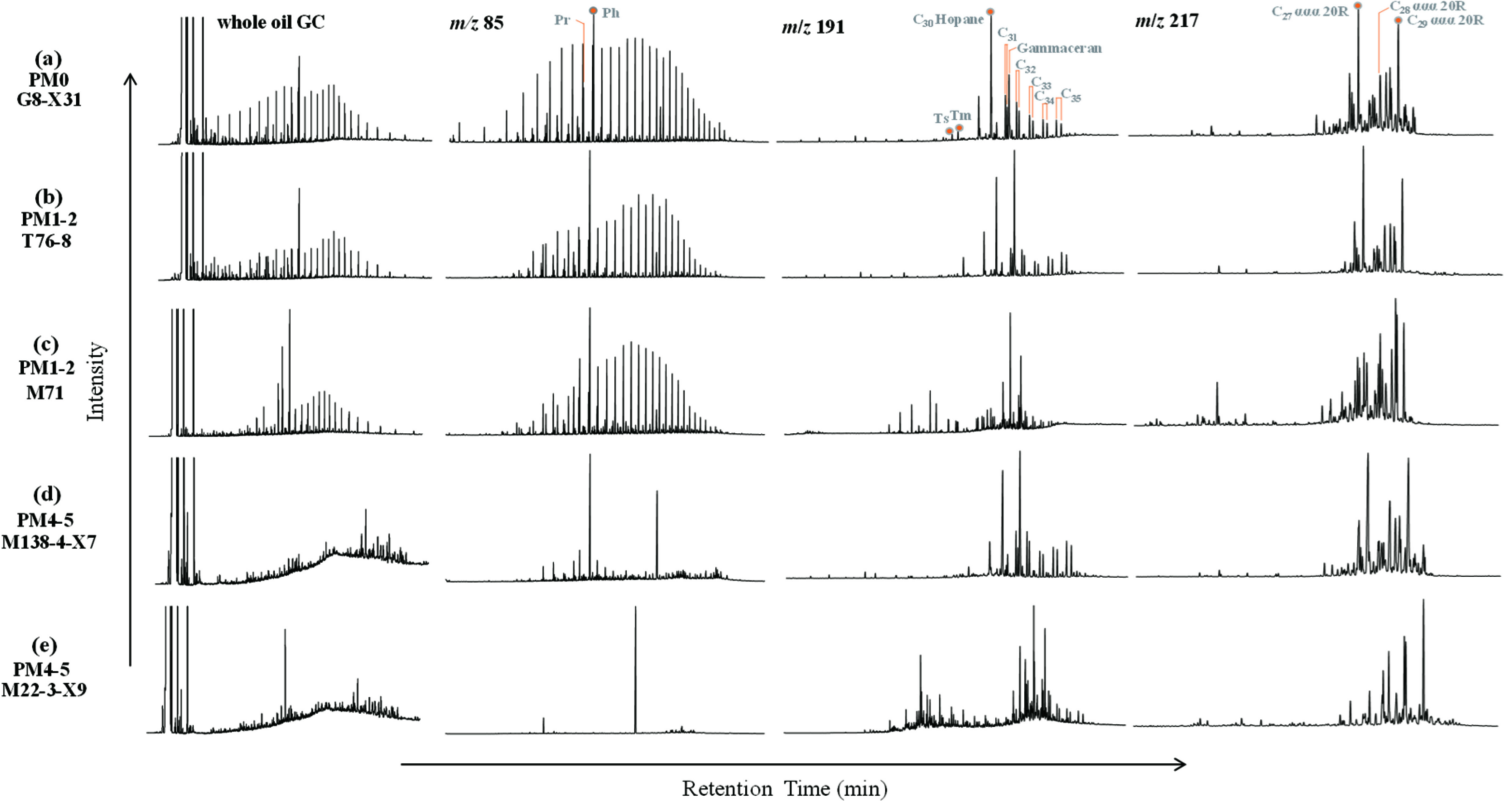
The typical GC traces of whole oils and molecular biomarker compositions of crude oils with different biodegradation levels:. Ph, phytane; Pr, pristane; Tm, trisnorhopane; Ts, trisnorneohopane.

Figure 2 and Table S1 (available at https://doi.org/10.5281/zenodo.15175535) show the typical GC traces of whole oil and mass chromatography of *n*-alkanes (*m/z* 85), terpanes (*m/z* 191), and steranes (*m/z* 217) for collected oils. In the Bamianhe Oilfield, *n*-alkanes in the northern oil from Well G8-X31 range from *n*-C_9_ to *n*-C_41_ with CPI of 0.96, Σ*n-*C_21−_/Σ*n-*C_22+_ of 0.49 and Pr/Ph of 0.28 (Table 1). Compared to the northern oil, the southern oils from Wells CX52, M138-5-X2, M22-3-X9, and M138-4-X7 are significantly depleted in *n*-alkanes, resulting in the unavailability of CPI and Σ*n-*C_21−_/Σ*n-*C_22+_, and presenting lower values of Pr/Ph and Ga/C_30_ H (Table 1). The similar distribution pattern of *n*-alkanes can be observed on oil samples from the Henan Oilfield. However, no significant variation can be detected on Ga/C_30_ H, C_19_–C_23_TT/C_30_H, Ts/(Ts + Tm), C_29_ ααα 20S/(20S + 20R), and C_29_ ββ/(αα + ββ) for oil samples from the same area (Table 1).

In the Jianghan Oilfield, compared to oil samples from Wells Z112-X27 and M36-X2-5, the oil samples from Wells M71 and T76-8 are depleted in <*n* C_16_ by presenting the lower Σ*n-*C_21−_/Σ*n-*C_22+_ ratio. The CPI value is in the range of 0.80–1.01 (Table 1). The ratios of Pr/Ph, C_19_–C_23_TT/C_30_H, Ts/(Ts + Tm), C_29_ ααα 20S/(20S + 20R), and C_29_ ββ/(αα + ββ) are relatively greater for oils from Wells M71 and M36-X2-5 relative to those for oils from the other wells. The opposite can be detected on the parameters of Ga/C_30_H (Table 1).

### Biodegradation levels of crude oils

**Fig 3:**
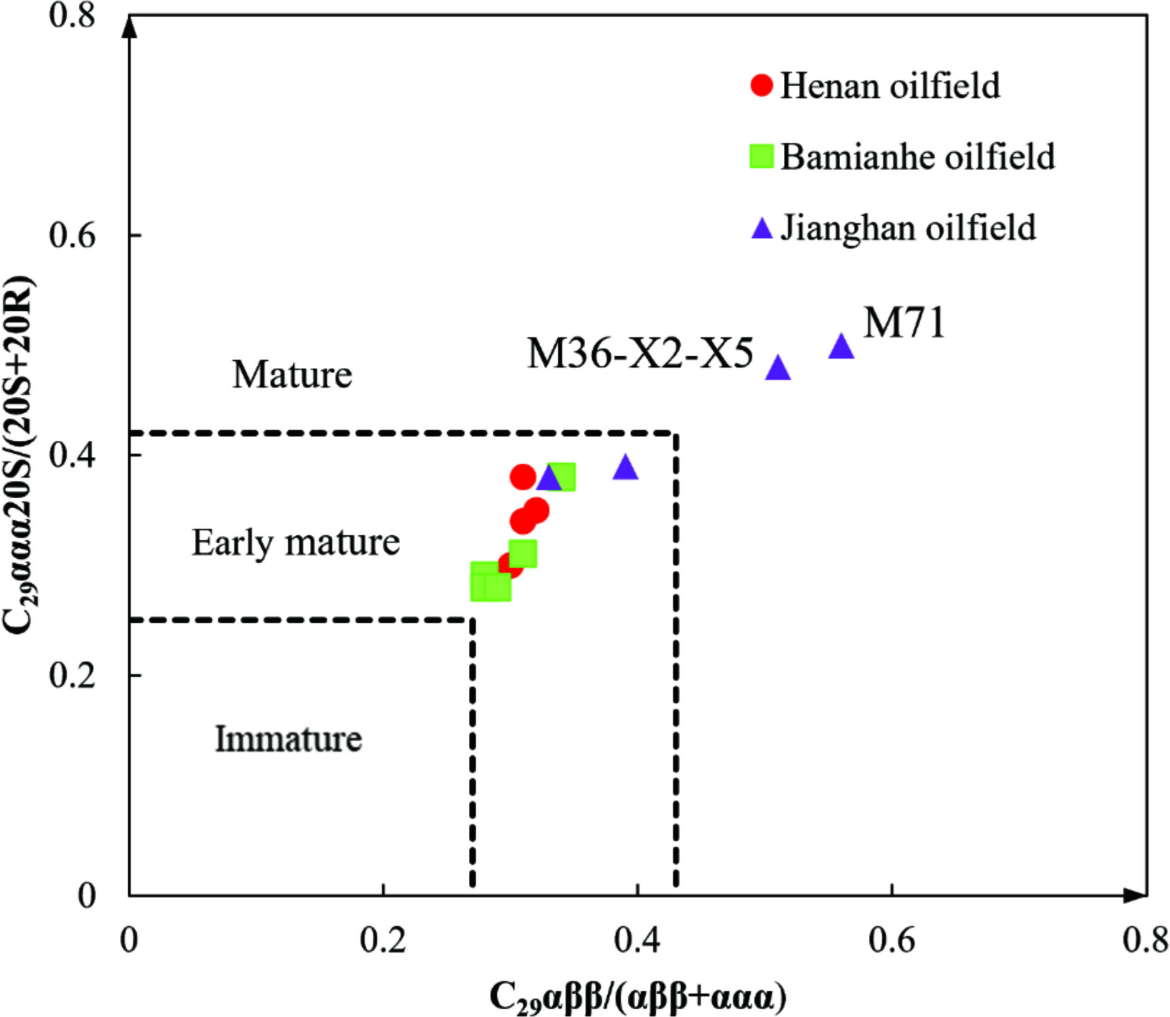
Correlation between the C_29_αββ/(αββ + ααα) and C_29_ααα20S/(20S + 20R) ratios of oils in the three oilfields.

The crossplot of C_29_-ββ/(αα + ββ) versus C_29_-ααα−20S/(20S + 20R) suggests that most collected oils are the early matured oil (Fig. 3), while oils from Wells M71 and M36-X2-5 are matured oils (40), which are consistent with the greater thermal maturity parameters of C_19_–C_23_TT/C_30_H, Ts/(Ts + Tm), C_29_ ααα 20S/(20S + 20R), and C_29_ ββ/(αα + ββ). The lower value of Ga/C_30_H for oils from these two wells indicates the relatively lower salinity of the water column during oil source deposition (41, 42). The sources of collected oil samples were deposited in the reduced anoxic environment as indicated by the low Pr/Ph of <1.0 for the normal oils (41, 42). The Pr/Ph ratio for biodegraded oils cannot be calculated due to the preferential consumption.

Oil biodegradation results in distinct distribution patterns of molecular biomarkers in oil samples from three oilfields. According to the biodegradation scales of Peters and Moldowan 25 (PM), the biodegradation level of collected oil samples is PM = 0–5. This is suggested by the distribution patterns of *n*-alkanes, since at PM = 1–5 *n*-alkanes are the most preferred targets in crude oil for microbial organisms (25). Accordingly, these oil samples can be classified into three groups:

1. Norman oils without biodegradation (PM = 0). These oils include oil samples from Wells G8-X31, M36-X2-5, and Z112-X27. These samples are enriched in *n*-alkanes as indicated by the whole oil GC traces (Fig. 2a; Fig. S1a and b). Terpanes and steranes are also well preserved (Fig. 2a; Fig. S1a and b; Table 1). The greater ratio of Ga/C_30_H for oil from Well Z112-X27 is attributed to the higher salinity during organic matter deposition in the water column (41, 42). The thermal maturity parameters including C_19_–C_23_TT/C_30_H, Ts/(Ts + Tm), C_29_ ααα 20S/(20S + 20R) and C_29_ ββ/(ββ + αα) indicate the early mature oil in the northern Bamianhe and Jianghan oilfields (Fig. 3b), which is consistent with previous investigations (43).
2. Lightly biodegraded oils (PM = 1–2). Oils from Wells T76-8 and M71 present a slight loss of *n*-alkanes as indicated by the lower CPI and Σ*n*C_21−_/Σ*n*C_22+_ ratios relative to those from Wells M36-X2-5 and Z112-X27. However, no significant alteration can be observed on isoprenoids, terpanes, and steranes (Fig. 2b and c; Table 1). The lower Pr/Ph and greater Ga/C_30_H for oils from Wells Z112-X27 and T76-8 indicate the associated source rocks were deposited in a more reduced water column with higher salinity (41, 42).
3. Heavily biodegraded oils (PM = 4–5). Oils from Wells HN1–4, CX52, M138-5-X2, M22-3-X9, M138-4-X7, and J5-X257 are significantly depleted in *n*-alkanes relative to the unaltered oils, and thus, the ratios of CPI, Σ*n*C_21−_/Σ*n*C_22+_, and Pr/Ph cannot be available (Table 1).Significant biodegradation presents the hump for unresolved complex matters (UCMs) on the whole oil GC traces (Fig. 2d and e; Fig. S1c through i). According to the biodegradation scales of Peters and Moldowan 25, the oil biodegradation level is assumed to be PM = 4–5.

### Microbial compositions of crude oils

To characterize prokaryotic and fungal communities in crude oil, we isolated DNA from oil samples, amplified and sequenced the 16S rRNA gene and the ITS gene. After filtering and trimming low-quality reads, we obtained 791,688 valid sequences of prokaryotic microorganisms and 560,584 fungal sequences for subsequent analyses.

Substantial differences between prokaryotes and fungi in crude oils can be observed at the phylum, family, and genus levels (see Fig. S2 to S4 at https://doi.org/10.5281/zenodo.15175535). At the phylum level, the prokaryotes mainly comprised the phylum Bacteroidota, Deferribacterota, Desulfobacterota, Firmicutes, and Proteobacteria (Gamma) (Fig. S2a). In contrast, fungi were predominated by Ascomycota and Basidiomycota (Fig. S3a). At the genus level, the prokaryotes were dominated by *Lentimicrobium* and *Tepidiphilus* in oils from Henan Oilfield (Fig. S4a), *Ignavibacterium*, *KCM-B-112*, *Klebsiella*, *Lactiplantibacillus*, *Marinobacter*, *Roseovarius* in oils from Bamianhe Oilfield, and *Desulfuromonas*, *Flexistipes*, *Halomonas*, *Marinobacter*, *Sulfurimonas* in oils from Jianghan Oilfield, respectively.

The fungal community was dominated by *Aspergillus* in oils from Henan Oilfield*, Aspergillus*, *Paraphoma*, *Pseudallescheria* in oils from Bamianhe Oilfield, and *Aspergillus*, *Meyerozyma* in oils from Jianghan Oilfield, respectively (Fig. S4b).

### Diversity analysis of microbial compositions in crude oils

**Fig 4:**
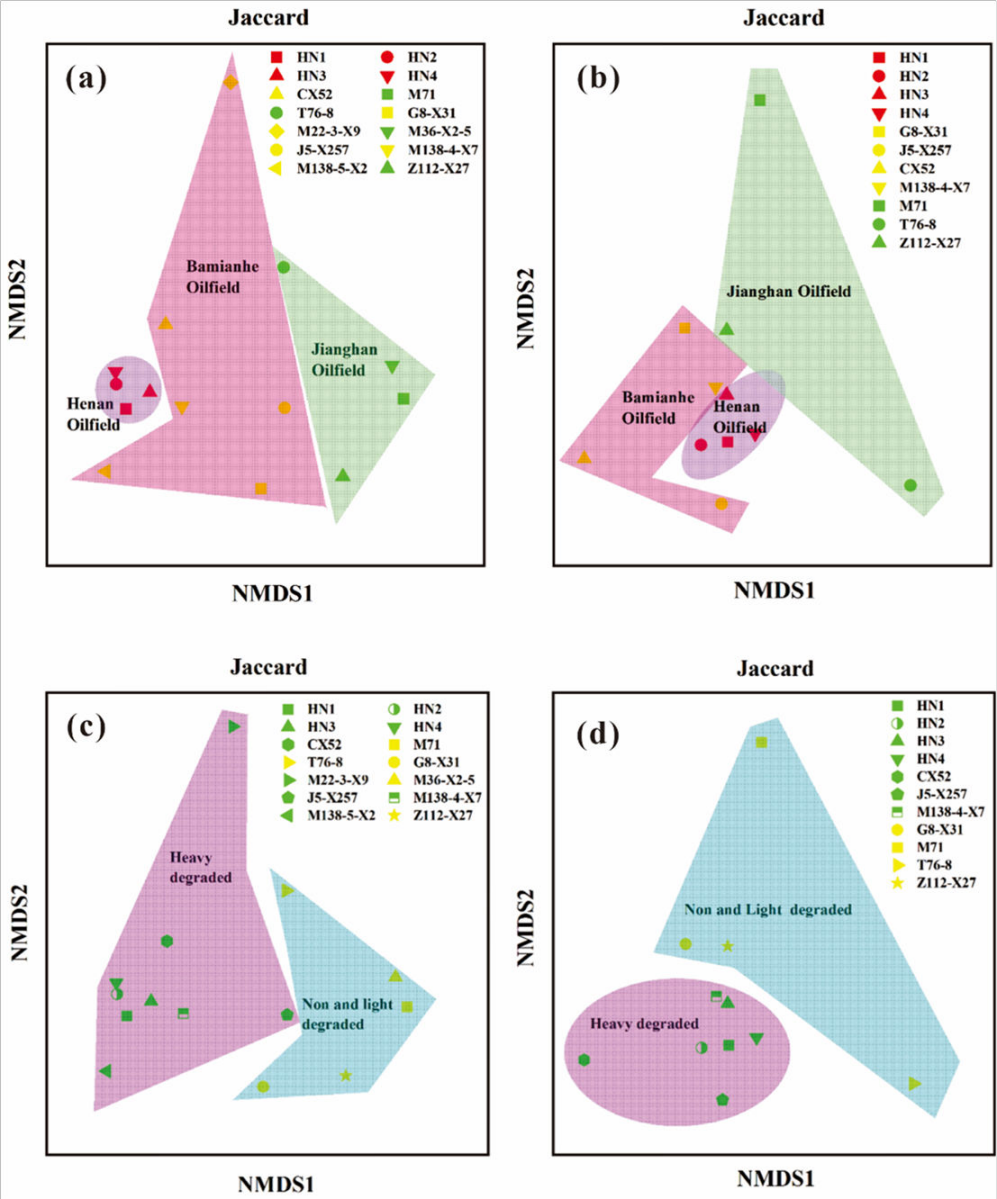
NMDS analysis of prokaryotic and fungal community structure in three oilfields and different degradation degrees of crude oils. NMDS shows the relationship among three oilfields and different biodegradation levels that these crude oil samples have significant differences. (a,c) Prokaryotic communities; (b,d) Fungal communities. Plots in (a–d) were calculated based on Jaccard distance.

The community structures of prokaryotes and fungi were closely related to the location of the study area and the oil biodegradation levels as indicated by the NMDS analysis. On the Jaccard crossplots, crude oils were classified into different groups in accordance with the oilfield location and oil biodegradation levels (Fig. 4). Bray-Curtis results also showed that prokaryotic communities varied significantly across different regions and oil biodegradation levels, but no significant differences were observed in fungal communities (see Fig. S5 and S10 at https://doi.org/10.5281/zenodo.15175535).

**Fig 5:**
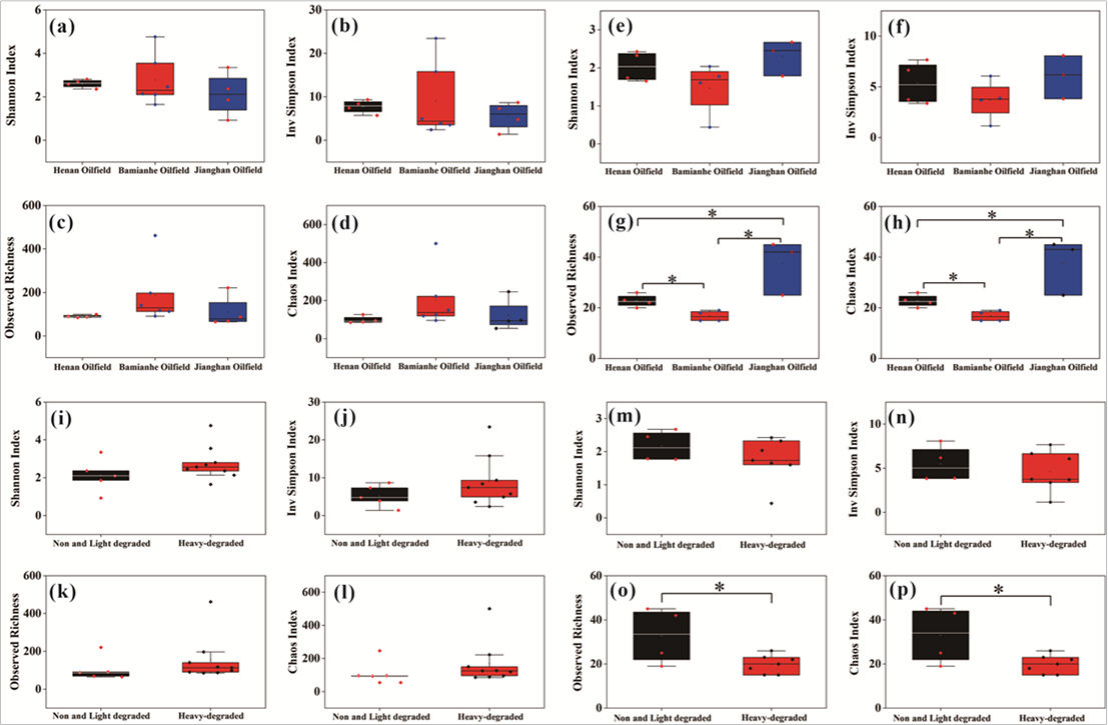
Analysis of prokaryotic and fungal community diversity in three regions and different levels of degradation of crude oils. The highest diversity of prokaryotic communities was found in the Bamianhe oilfield. The highest diversity of fungal communities was found in the Jianghan oil field, especially the Shannon and Inv Simpson index differed significantly. The diversity of heavily degraded prokaryotic communities was greater than that of non-degraded and lightly degraded prokaryotic communities. The diversity of non-degraded and lightly degraded fungal communities was higher than that of heavily degraded fungal communities, and there were significant differences among samples. Prokaryotes community (a–d, i–l); Fungi community (e–h, m–p); *indicates *P* < 0.05.

The Shannon index, Inv Simpson index, Observed richness, and Chao1 index were calculated to compare the diversity of prokaryotic and fungal communities in crude oils (Fig. 5). Four indices demonstrated that the diversity of prokaryotic communities was highest in oils from Bamianhe Oilfield, and the diversity of fungal communities was highest in oils from Jianghan Oilfield (Fig. 5a through d). More interestingly, heavily biodegraded oils presented greater diversity in prokaryotes, but lower diversity in fungi than normal and slightly biodegraded oils (Fig. 5i through l).

### Microbial ASV analysis based on the locality of crude oils

**Fig 6:**
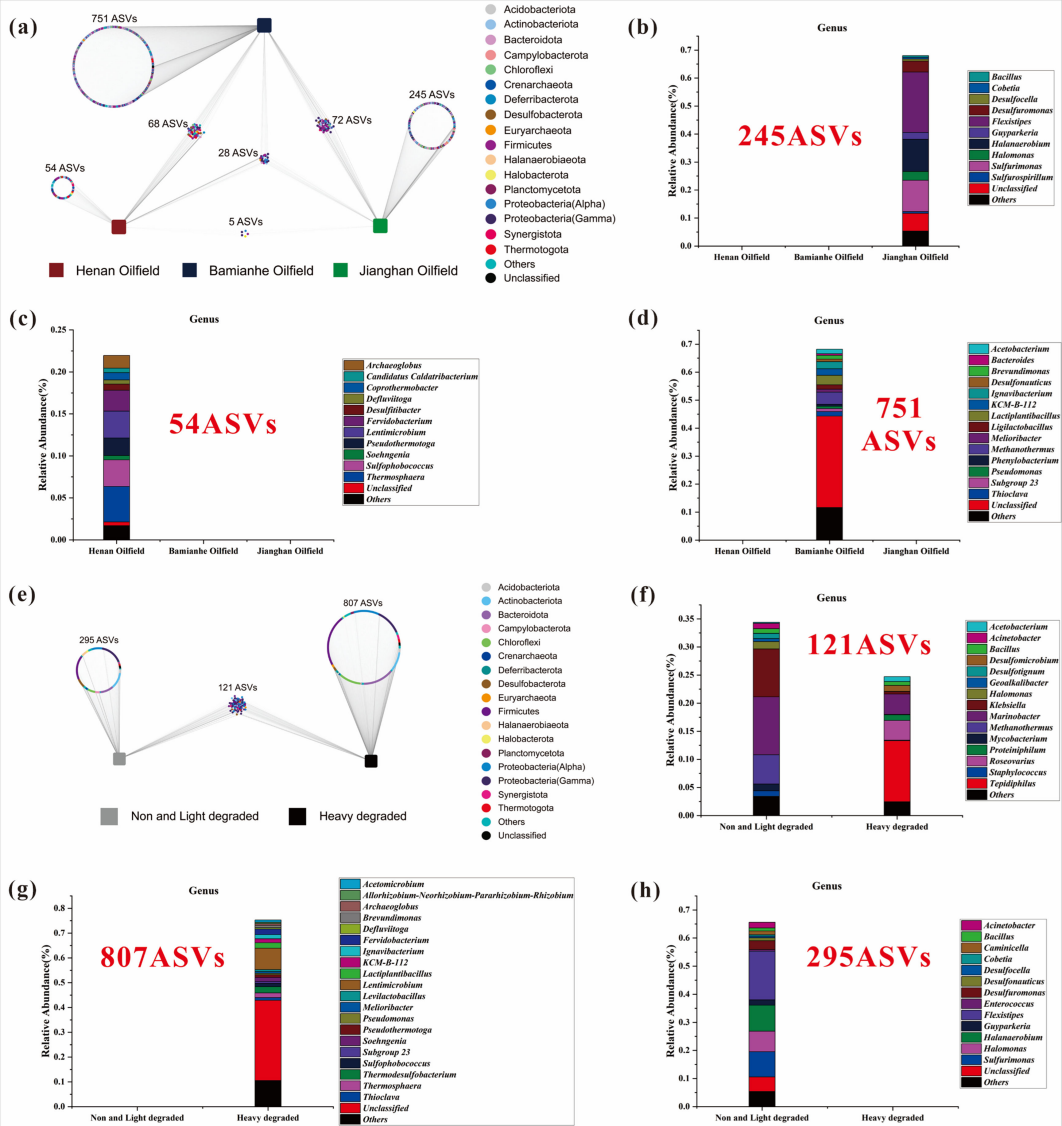
ASV results of prokaryotic communities compared with oil samples from three oilfields and different degrees of degradation. (a) ASV results of three oilfields at phylum level; (b) Jianghan Oilfield particular prokaryotes at genus level; (c) Henan Oilfield particular prokaryotes at genus level; (d) Bamianhe Oilfield particular prokaryotes at genus level; (e) two kinds of degradation phylum level ASV results; (f) the two kinds of degradation degree shared prokaryotes at genus level; (g) heavily degraded samples particular prokaryotes at genus level; and (h) non-biodegraded and lightly biodegraded samples particular prokaryotes at genus level.

According to the locality of collected oil samples, we can explore the unique and shared prokaryotes and fungi in oils from these three oilfields based on ASV analysis (Fig. 6a through d) and (see Fig. S15 to S17 at https://doi.org/10.5281/zenodo.15175535). The relevant results are stated as follows.

#### Prokaryotes

The results focused on the relatively high abundance genera (>10%). Specifically, oils collected from Bamianhe Oilfield have the representative genera including *Methanothermus*, *Lactiplantibacillus*, and *Ignavibacterium* (Fig. 6d). Oils from Jianghan Oilfield contain the unique genera including *Sulfurimonas*, *Halanaerobium*, and *Flexistipes* (Fig. 6b). Oils from Henan Oilfield have the lowest abundance of unique prokaryotic ASVs and the genera including *Thermosphaera*, *Sulfophobococcus*, and *Lentimicrobium* (Fig. 6c).

The shared genera are 68 ASVs in oils from Henan and Bamianhe oilfields (*Thermodesulfobacterium*, *Soehngenia*, *Longilinea*, *LentimicrobiumJGI-0000079-D21*, *Fervidobacterium*, *Desulfomicrobium*, *Defluviitoga*, *Allorhizobium-Neorhizobium-Pararhizobium-Rhizobium*, and *Acetomicrobium* ) (see Fig. S16d at https://doi.org/10.5281/zenodo.15175535), 5 ASVs in oils from Henan and Jianghan oilfields (*Pseudomonas*, *Pseudoalteromonas*, *Methylobacterium-Methylorubrum*, *Brevundimonas*, and *Bacillus*) (see Fig. S16b at https://doi.org/10.5281/zenodo.15175535), and 72 ASVs in oils from Bamianhe and Jianghan oilfields (*Roseovarius*, *Marinobacter*, *Klebsiella*, *Halomonas*, *Geoalkalibacter*, *Desulfotignum*, *Caminicella*, *Bifidobacterium*, *Bacillus*, and *Acinetobacter*) (see Fig. S16c at https://doi.org/10.5281/zenodo.15175535).

The prokaryotic communities in oils can be clustered into 28 ASVs, and the shared genera (>1%) were composed of *Tepidiphilus*, *Proteiniphilum*, and *Mycobacterium* (see Fig. S16a at https://doi.org/10.5281/zenodo.15175535).

#### Fungi

The fungal communities in oils from Henan Oilfield can be clustered into 29 ASVs. And the results focused on the relatively high abundance genera. The unique fungal communities with high relative abundance (>0.7%) include *Pseudolagarobasidium*, *Fomitopsis*, and *Cadophora* (Fig. S15c). Similarly, the fungal communities in oils from Bamianhe Oilfield can be clustered into 22 ASVs. The unique fungal communities include *Pseudallescheria*, *Paraphoma*, and *Aspergillus* (see Fig. S15d at https://doi.org/10.5281/zenodo.15175535). Although the fungal communities in oils from Jianghan Oilfield can be clustered into 59 ASVs, the unique fungal communities include *Porostereum*, *Meyerozyma*, and *Cerrena* (see Fig. S15b at https://doi.org/10.5281/zenodo.15175535).

The shared fungal communities were one ASV for oils from Henan and Bamianhe oilfields (*Hymenochaete*) (see Fig. S17d at https://doi.org/10.5281/zenodo.15175535), seven ASVs for oils from Henan and Jianghan oilfields (*Phlebia*, *Penicillium*, *Malassezia*, *Cladosporium*, and *Aspergillus* (see Fig. S17b at https://doi.org/10.5281/zenodo.15175535), and seven ASVs for oils from Bamianhe and Jianghan oilfields (*Tomentella*, *Phlebia*, *Phaeophleospora*, *Peroneutypa*, *Peniophora*, and *Gloeostereum*) (see Fig. S17c at https://doi.org/10.5281/zenodo.15175535), respectively. The shared 13 ASVs for oils from these three oilfields comprise *Sterigmatomyces*, *Lyomyces*, *Cladosporium*, *Aspergillus*, *Alternaria* (see Fig. S17a at https://doi.org/10.5281/zenodo.15175535).

### Microbial ASV analysis based on oil biodegradation levels

According to oil biodegradation levels, the distinct and equivalent communities of prokaryotes and fungi in oils from three oilfields were conducted by using ASV analysis (Fig. 6e through h and see Fig. S18 at https://doi.org/10.5281/zenodo.15175535). The relevant results are stated as follows.

#### Prokaryotes

The prokaryotic community for heavily biodegraded oils can be clustered into 807 ASVs, and the genera with relatively abundance >2% include *Thermodesulfobacterium*, *Lentimicrobium*, and *Lactiplantibacillus* (Fig. 6g). The prokaryotic community in non-biodegraded and lightly biodegraded oils can be clustered into 295 ASVs, and the genera with relatively high abundance >2% include *Sulfurimonas*, *Halanaerobium*, and *Flexistipes* (Fig. 6h). The shared 121 ASVs of the prokaryotic community and the genera with relatively abundance >2% include *Tepidiphilus*, *Marinobacter*, and *Klebsiella* (Fig. 6f).

#### Fungi

The fungal community in heavily biodegraded oils can be clustered into 47 ASVs, and the genera with relatively abundance >1% were *Pseudallescheria*, *Paraphoma*, and *Aspergillus* (see Fig. S18c at https://doi.org/10.5281/zenodo.15175535). The fungal community in non-biodegraded and lightly biodegraded oils can be classified into 68 ASVs with the relatively high abundance genera (>0.8%) including *Phlebia*, *Phaeophleospora*, and *Meyerozyma* (Fig. S18b). In general, 23 ASVs in these two kinds of oils can be recognized, and the shared relatively high abundance genera (>2%) were *Sterigmatomyces*, *Cladosporium*, and *Alternaria* (see Fig. S18d at https://doi.org/10.5281/zenodo.15175535).

## DISCUSSION

### Prokaryotic communities indigenous to crude oil with varying biodegradation levels

Substantial differences in prokaryotic communities can be observed in oils with heavy and slight biodegradation levels (see Tables S11 and S13 at https://doi.org/10.5281/zenodo.15175535). This is caused by microbial activity under different native and artificial circumstances altered by the practice of thermal recovery. Reservoir temperature plays a critical role in regulating the microbial communities in crude oils and hence oil biodegradation levels. This is confirmed by the fact that oils hosted in the reservoirs with *T* < 80°C tend to be microbially degraded to varying degrees (44). Heavily biodegraded oils were hence more enriched in microorganisms than normal and slightly biodegraded oils (45). In this study, heavily biodegraded oils from Henan and the southern Bamianhe oilfields were produced from low-temperature petroleum reservoirs with *T* = 38–68°C (Table 1). While the unaltered and lightly biodegraded oils from Jianghan and the northern Bamianhe oilfields (G8-X31) were mainly produced from high-temperature petroleum reservoirs with *T* = 60–121°C (Table 1). The detailed differences in microbial compositions for oils with different biodegradation levels are presented below.

#### The unique prokaryotes in heavily biodegraded oils

NMDS and related statistics (MRPP, ANOSIM, and PERMANOVA) indicated significant differences in prokaryotic communities in crude oils with different biodegradation levels (*P* < 0.05) (see Tables S11 and S13 at https://doi.org/10.5281/zenodo.15175535). This is generally consistent with the previous study by Sierra-Garcia et al. 46, which demonstrated that bacterial communities in biodegraded oils are more diverse than those in unaltered oils (46). A series of unique prokaryotic communities was discovered in heavily biodegraded oils, including *Thioclava*, *Thermosphaera*, *Sulfophobococcus*, *Soehngenia*, *Fervidobacterium*, and *Archaeoglobus* (Fig. 6g)

The *Thioclava* genus was detected in oil-polluted seawater of the Yellow Sea as crude oil-degrading bacteria (47). It is hence not surprising to find them in oils with PM 4–5 from Henan and Bamianhe oilfields. The members of *Archaeoglobus* were hyperthermophilic archaea and grew anaerobically at around 80°C (48) and were dominant in hydrocarbon degradation (49). They were detected in oils from Well HN1-4 in the Henan Oilfield (*T* = 38°C) and Well G8-X31 in the Bamianhe Oilfield (*T* = 121°C).

The members of *Sulfophobococcus* were found in hot springs (50), and the genus *Thermosphaera* was isolated from hot springs as hyperthermophilic archaea that survive at about 85°C (51). Both bacteria were found in heavily biodegraded oils from Henan Oilfield.

The members of *Fervidobacterium* were found to live in anaerobic and thermophilic hot springs at around 80°C (52) and have been detected in petroleum hydrocarbon-rich oil refinery sludge (53). They were found in oils produced from the Henan Oilfield (*T* = 38°C) and the Bamianhe Oilfield (*T* = 52–59°C).

Obviously, the low-temperature reservoir with *T* = 38.1–38.6°C in the Henan Oilfield and *T* = 52–68.3°C in the southern Bamianhe Oilfield is not suitable for the living of these prokaryotes. Their occurrence in the cool reservoir may be related to the practice of steam-assisted gravity drainage with the attempt to enhance heavy oil recovery in these regions. This also indicated that the saturated water steam injected into the heavy oil sandstone reservoirs may favor the reproduction of these thermophilic bacteria and hence change the native microbial community, except for Well G8-X31 in the Bamianhe Oilfield.

Bacteria of the genus *Soehngenia* were mesophilic/thermostable bacteria and present widely in heavy oil reservoirs around 50°C (54, 55). They were also detected in the heavy oils from the Henan and Bamianhe oilfields (*T* = 38.1–58.7°C) and may represent the native prokaryotes accompanied by heavy oil biodegradation in subsurface petroleum reservoirs.

#### The unique prokaryotes in non-biodegraded and lightly biodegraded oils

In unaltered and slightly biodegraded oils (PM 0–2), a series of unique bacteria were detected including *Caminicella*, *Desulfonauticus*, *Desulfocella*, and *Halomonas*. The temperature and water geochemistry of petroleum reservoirs are assumed to play the major role in regulating the occurrence of these prokaryotes.

The *Caminicella* genus was isolated from hydrothermal vents such as East-Pacific Rise (56) and formation water produced from hot petroleum reservoir with *T* = 131°C (57). The *Desulfonauticus* genus, thermophilic and hydrophilic sulfate-reducing bacteria, was also detected in hydrothermal vents of East-Pacific Rise at 2,600 m (58) and produced formation water from Northern Germany Oilfield (59). They can survive within the high-temperature regimes and were only detected in oil from Well G8-X31 in the northern Bamianhe Oilfield with *T* = 120°C (Table 1). This highlights the critical importance of reservoir temperature in controlling the occurrence of these prokaryotes. More importantly, the occurrence of *Desulfonauticus* reminds us of the possible risk of hydrogen sulfide generated by this genus.

*Desulfocell*a genus was a group of halophilic sulfate-reducing bacteria and usually discovered in salt lake sediments, such as Great Salt Lake (60). This genus is only detected in oils from Well M36-X2-5 in Jianghan Oilfield. Moreover, abundant *Halomonas* genus was also detected in oils from the Jianghan Oilfield, which have been found in the sediment with high salinity (61). The occurrence of these two genera generally agrees with the natural geological environments of the Jianghan Basin. It is well known that the Jianghan Basin was a typical salt lake during the Eocene period with the deposition of thick salt sequences (17). The saturated formation water contains abundant sulfates serving as terminal electron acceptors to drive the sulfate-reducing metabolism of *Desulfocella* spp. Except for *Desulfocella* and *Halomonas,* a large number of halophilic microorganisms were detected in oils from the Jianghan Oilfield, including the genera *Flexistipes*, *Guyparkeria*, *Halanaerobium*, and *Cobetia*. As stated above, the high salinity of formation water in the Jianghan Oilfield should be responsible for this.

#### The shared prokaryotes in collected oils

Regardless of biodegraded levels, several common prokaryotes can be detected in collected oil samples including *Marinobacter*, *Klebsiella*, *Desulfomicrobium*, *Bacillus*, *and Acinetobacter*. Previous investigations have demonstrated that these genera express the capability of degrading oil (62–66). Although the structure of dominant bacterial communities varies with hydrocarbon compositions (67), the shared prokaryotes occurring in oils with varying biodegradation levels indicate their highly adaptable and critical role in carbon cycling on the Earth. More investigations are hence required here to make a better understanding, which is beyond the scope of this study.

### Fungi communities indigenous to crude oil with varying biodegradation levels

Unlike prokaryotes, fungi show low diversity in heavily biodegraded oils relative to that in unaltered and slightly biodegraded oils (Fig. 5m through p). This may be attributed to the chemical compositions of crude oils, since fungi prefer to metabolize *n*-alkanes and prokaryotes were prone to consuming branched and aromatic compounds (68). The enrichment of *n*-alkanes in unaltered and slightly degraded oils was feasible for the living of fungi and hence higher diversity. The opposite was true for fungi in severely biodegraded oils due to the depletion of *n*-alkanes. The unique and shared fungi in oils with different biodegradation levels are presented as the following.

#### The unique fungi heavily biodegraded oils

Fungi were the second-best oil-degrading organisms relative to bacteria (19). Abundant *Aspergillus* was found in heavily degraded oil samples from Henan and Bamianhe oilfields, which have been reported to degrade petroleum hydrocarbons (69). Besides the genus *Coriolopsis* (70), *Pseudolagarobasidium* (71), and *Pleurotus* (72) can secrete laccase, lignin peroxidase, and manganese peroxidase essential enzymes, and the genus *Cadophora* was capable of degrading oil alkanes (73). All genera can be detected in Henan Oilfield. A consortium of three enzymes showed a significant level of enzyme activity to degrade hydrocarbons (74). The genera *Pseudallescheria* (75), *Paraphoma*, and *Coprinellus* (73) were capable of degrading oil alkanes, and the related members were detected at petroleum-contaminated sites (e.g., soils). They were also detected in heavily biodegraded oils from Bamian Oilfield (J5-X257 well, *T* = 68℃). The genus *Ceriporia* was detected in severely degraded oils from Bamianhe Oilfield (M138-4-X7 well, *T* = 58℃), and its related members can degrade PAHs (76). Due to the practice of steam-assisted oil drainage in these heavy oil reservoirs, the occurrence of these unique fungi indicates that the artificially altered environments of petroleum reservoirs may be suitable for the living of these fungi.

#### The unique fungi in non-degraded and lightly biodegraded oils

The unique fungal community in non-degraded and lightly degraded crude oil samples mainly consisted of the genera *Alternaria*, *Aspergillus*, *Coprinopsis*, *Cerrena*, *Peniophora*, *Meyerozyma*, and *Phlebia*. The genera *Alternaria* and *Aspergillus* have been reported to use crude oil as a carbon and energy source (69). The genera *Coprinopsis* (77), *Cerrena* (78), and *Peniophora* (79) can produce laccases during the degradation of petroleum hydrocarbons (80). The yeast *Meyerozyma* has been reported to degrade petroleum hydrocarbons by using *n*-alkanes as its sole source of carbon and energy (81). These organisms were detected mainly in non-degraded and lightly degraded oil samples from Jianghan Oilfield. As mentioned above, this may be closely related to the native environments of petroleum reservoirs, including the high salinity of formation water and chemical compositions of crude oils. And the genus *Phlebia* was detected in Bamianhe and Jianghan oilfields, can degrade polycyclic aromatic hydrocarbons (PAHs), and co-cultivation with related bacteria will have a higher degradation rate (82). So it is assumed that the native environments of petroleum reservoirs, including the temperature and high salinity of formation water and microbial interactions, influence this genus. In order to obtain more accurate evidence, we will further analyze the correlation between the environment, microorganisms, and microbial interactions.

#### The shared fungi in collected oils

The shared fungal communities include genus *Aspergillus*, *Cladosporium*, and *Sterigmatomyces*. Fungi were more resistant to harsh environmental conditions than bacteria (83). Therefore, we speculate that these fungi can widely adapt to the different environments of three oilfields. Members of the genus *Sterigmatomyces* were reported to have been found under high-salt conditions (84). Further research is needed to unravel the genus *Sterigmatomyces* and its implications for crude oil degradation.

### Conclusions

This study presents the detailed prokaryotic and fungal communities in crude oils from three oilfields in China. The main points present as the following:

1. Compared to oils from the Jianghan Oilfield and the north of Bamianhe Oilfield, oils from the Henan Oilfield and the south of Bamianhe Oilfield suffer from moderate biodegradation. Most oils were the early matured oil, and oils from Well M71 and M36-X2-5 of Jianghan Oilfield were matured oil. The oil sources were deposited in the reduced anoxic environments.
2. The oil extraction technology and the natural environments of petroleum reservoirs, including reservoir temperature and formation water salinity, are revealed as important factors on prokaryotic communities in crude oils. And the statistical analysis revealed significant variations of prokaryotic communities (*P* < 0.05) within three oilfields and different biodegradation levels of oils. The diversity analysis also revealed that heavily degraded prokaryotic communities were greater than that of non-degraded and lightly degraded prokaryotic communities. Moreover, the thermophilic prokaryotes including *Thioclava*, *Thermosphaera*, *Sulfophobococcus*, *Soehngenia*, *Fervidobacterium*, and *Archaeoglobus* were detected mainly in biodegraded heavy oils produced by steam-assisted gravity drainage in the Henan Oilfield and the southern Bamianhe Oilfield. The halophilic prokaryotes were detected mainly in oils from sandstone reservoirs containing hypersaline formation water from Jianghan Oilfield, such as *Desulfocella*, *Halomonas*, *Flexistipes*, *Guyparkeria*, *Halanaerobium*, and *Cobetia*.
3. The oil biodegradation levels have significantly affected the fungi communities in crude oils. The statistical analysis revealed significant variations of fungal communities (*P* < 0.05) within three oilfields and different biodegradation levels of oils. And the diversity analysis revealed that non-degraded and lightly degraded fungal communities were higher than that of heavily degraded fungal communities. The unique fungi in heavily biodegraded oils include *Aspergillus*, *Coriolopsis*, *Pseudolagarobasidium*, *Pleurotus*, *Cadophora*, *Pseudallescheria*, *Paraphoma*, *Coprinellus*, and *Ceriporia*. The unique fungi in non-degraded and lightly biodegraded oils include *Alternaria*, *Aspergillus*, *Coprinopsis*, *Cerrena*, *Peniophora*, *Meyerozyma*, and *Phlebia*. Moreover, as it is concerned with the general living environment of fungi, the correlation between the environment, microorganisms, and microbial interactions must be considered.

These enlightening results suggest that the diversity of prokaryotes and fungi communities of endogenous crude oils and the affecting factors of different crude oils microbial communities are significant for enhanced oil recovery.

## Data Availability

Raw sequencing reads of all samples were deposited in the NCBI database (http://www.ncbi.nlm.nih.gov/) under the BioProject accession numbers PRJNA1020134 for the prokaryotic community data sets and PRJNA1020147 for the fungal community data sets.
